# Nature’s Plastic Predators: A Comprehensive and Bibliometric Review of Plastivore Insects

**DOI:** 10.3390/polym16121671

**Published:** 2024-06-12

**Authors:** Joseph Boctor, Gunjan Pandey, Wei Xu, Daniel V. Murphy, Frances C. Hoyle

**Affiliations:** 1 Bioplastics Innovation Hub, Food Futures Institute, Murdoch University, Murdoch, WA 6150, Australia; w.xu@murdoch.edu.au (W.X.); daniel.murphy@murdoch.edu.au (D.V.M.); fran.hoyle@murdoch.edu.au (F.C.H.); 2 Commonwealth Scientific and Industrial Research Organisation (CSIRO), Environment, Acton, ACT 2601, Australia; gunjan.pandey@csiro.au; 3 SoilsWest, Centre for Sustainable Farming Systems, Food Futures Institute, Murdoch University, Murdoch, WA 6150, Australia

**Keywords:** plastic biodegradation, mineralisation, polymers, exaptation

## Abstract

Unprecedented plastic production has resulted in over six billion tons of harmful waste. Certain insect taxa emerge as potential agents of plastic biodegradation. Through a comprehensive manual and bibliometric literature analysis, this review analyses and consolidates the growing literature related to insect-mediated plastic breakdown. Over 23 insect species, representing Coleoptera, Lepidoptera, and 4 other orders, have been identified for their capacity to consume plastic polymers. Natural and synthetic polymers exhibit high-level similarities in molecular structure and properties. Thus, in conjunction with comparative genomics studies, we link plastic-degrading enzymatic capabilities observed in certain insects to the exaptation of endogenous enzymes originally evolved for digesting lignin, cellulose, beeswax, keratin and chitin from their native dietary substrates. Further clarification is necessary to distinguish mineralisation from physicochemical fragmentation and to differentiate microbiome-mediated degradation from direct enzymatic reactions by insects. A bibliometric analysis of the exponentially growing body of literature showed that leading research is emerging from China and the USA. Analogies between natural and synthetic polymer’s degradation pathways will inform engineering robust enzymes for practical plastic bioremediation applications. By aggregating, analysing, and interpreting published insights, this review consolidates our mechanistic understanding of insects as a potential natural solution to the escalating plastic waste crisis.

## 1. Introduction

Natural polymers like beeswax, rubber, and silk have contributed significantly to human civilization and industries throughout history [1,2]. A pivotal moment was Leo Baekeland’s invention of Bakelite in 1907, enabling widespread plastic polymer use including nylon thereafter [3,4]. However, the negative impacts of plastics emerged in 1960, with the first reported marine species ingesting plastic debris, marking humanity’s influence on the Anthropocene [5,6]. Global plastic production then exponentially increased since the 1990s [7,8], resulting in over 10-fold more plastic waste than human biomass [9,10]. This threatens marine ecosystems, with more than 1400 species documented ingesting plastic debris, leading to health issues and mortality [11]. Microplastics exacerbate environmental contamination as carriers for pollutants like polychlorinated biphenyls (PCBs), pesticides, and polycyclic aromatic hydrocarbons (PAHs) that enter ecosystems and food chains, triggering adverse effects [12,13,14]. Ubiquitous plasticizers bisphenols and phthalates, for example, raise toxicity concerns associated with plastic migration into food/drink and links to conditions like obesity [15].

Insects, once viewed as nuisances damaging plastics, now offer a potential solution for plastic waste through biodegradation. Plastivore insects, recognized since the 1940s for their ability to consume plastic [16], present an alternative approach. For example, *Tribolium confusum* larvae significantly decreased the weight of polystyrene samples by 52% [17], indicating their potential for plastic degradation. Similar reports of plastic degradation have been documented across various insect species and polymer types [18]. While some insects depend on gut microbiota for plastic digestion, others repurpose independent lysis mechanisms [19,20]. Identifying specific enzymes responsible for plastic degradation remains a crucial area of investigation. Current research prioritizes efficiency in addressing plastic waste, focusing on optimizing existing enzymes rather than identifying new ones [21]. Despite limitations in biodegradation abilities, computational approaches offer promise for the accelerated evolution of plastic-degrading enzymes. Comparative genomics studies, like those on the greater wax moth, reveal the repurposing of enzyme families for plastic degradation [20,22] suggesting exaptation, rather than traditional evolution, as the dominant mechanism behind plastic-degrading enzymes. Further studies are crucial for enhancing the capabilities of insects and their enzymes in biodegrading plastics, potentially rendering insects invaluable allies in combating plastic waste. Here, we aim to analyse literature reports, employing manual and bibliometric curation extensively, exploring repurposed potential mechanisms, preconditioning natural substrates, and providing insight into employing insects to solve the plastic waste problem. While prior reviews have evaluated microbial enzymes in plastic degradation [17,18], this study comprehensively analyses the emerging role of insect-mediated breakdown. As higher organisms demonstrating plastic consumption abilities, insects remain poorly understood in terms of the enzymes and mechanisms underlying this ability. This review aims to consolidate current knowledge on insect plastic degradation while also identifying avenues for future research, such as elucidating the potential neofunctionalised pathways involved related to natural polymers digestion [18,22].

## 2. Literature Analysis

### 2.1. The Plastic Waste Problem: Statistical Magnitude and Threats

Global plastic manufacturing doubled from 2000 to 2019, reaching 460 million tonnes annually, contributing approximately 3.4% of greenhouse gas emissions [7]. Since the introduction of Bakelite, the global production of plastics has reached a staggering amount of over 8.3 billion tonnes [23]. Despite this, only approximately 9% of plastic has been recycled [24,25]. Incineration accounts for approximately 19%, emitting approximately 1.58 billion tonnes of carbon dioxide [7,26]. The remaining 72% (approximately 6 billion tonnes) either ended up in landfills or the environment, surpassing the total biomass weight of all humans combined by over 10 fold, estimated at 400 million tonnes in 2022 [7,9]. Moreover, an estimated 5.25 trillion plastic pieces are floating in the world’s seas, posing a significant risk to marine life and the food chain [27]. Plastic debris has inflicted harm on at least 1400 marine species [11]. Chemicals leaching from mismanaged plastics enter various food chains, contaminating organisms at all levels, including fish populations [28] and humans [13]. More concerningly, studies suggest that microplastics, at “environmentally comparable” concentrations, induce adverse effects such as cell death, immunological responses, genotoxicity, and oxidative stress [14].

**Figure 1:**
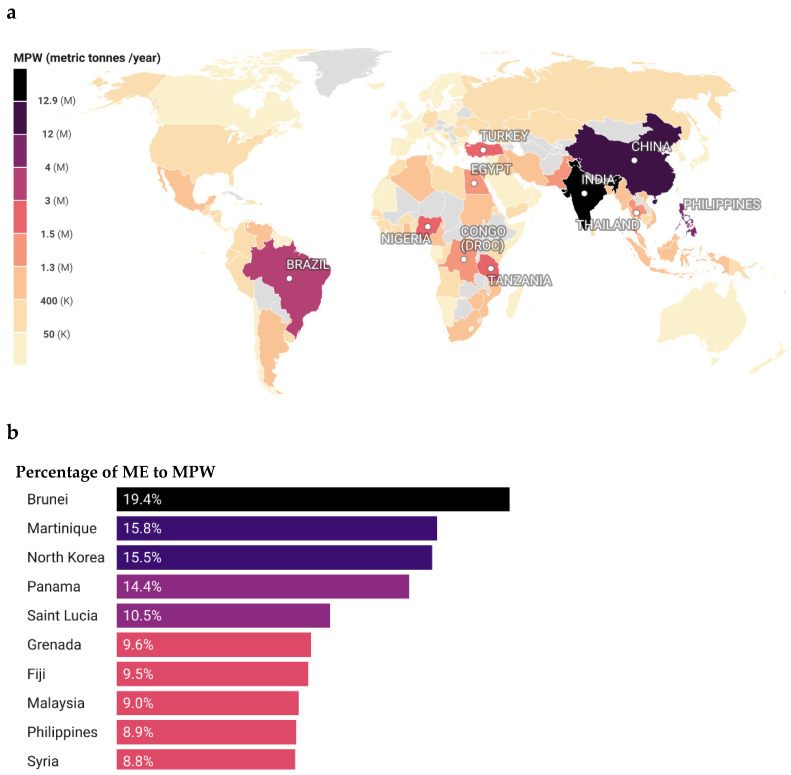
(**a**) Modelled global weight of mismanaged plastic waste (MPW) generated in metric tonnes annually. Top 10 countries in MPW generation are labelled. (**b**) Countries recording the highest percentage of riverine plastic waste emissions entering the oceans (ME) as a percentage of the total MPW produced. Countries with unavailable data are greyed out. Data source is (Meijer et al. 2021) [29].

Based on the research conducted by Meijer et al. (2021) on plastic waste transport from river basins to oceans, utilizing data from 163 countries and over 1000 rivers [29], we created visual representations of the top 10 countries, with the highest mismanaged plastic waste (MPW) in metric tonnes per year (Figure 1a) and the ratio of MPW reaching oceans from river basins, termed as river source plastic mass emissions (ME) (Figure 1b). Further analysis of average population data revealed the Democratic Republic of Congo had the highest MPW per capita at 253 kg/capita/year, followed by Comoros at 68 kg/capita/year and Trinidad and Tobago at 60 kg/capita/year (Table S1, Supplementary Materials) [29,30]. An important contextual factor when interpreting the results is the estimated 2% of global plastic waste that is internationally traded between countries [7]. Some countries, commonly import plastic scrap for recycling purposes, seeking related economic benefits [7]. With less than 1% of global plastic production currently biodegradable or biobased, adopting alternatives to conventional plastics faces infrastructure, financial, and property limitations [31]. This highlights the urgent need for eco-friendly solutions to address the mounting plastic waste crisis, necessitating a comprehensive strategy including recycling promotion, reduced usage, replacement, and reuse. Biodegradation emerges as a viable option, converting plastics into valuable materials. Scientists identifying organisms or enzymes for bio-cycling primarily focus on microbes, fungi, and insects. Computational screening unexpectedly revealed plastic-degrading enzymes even in human saliva metagenomes [32], suggesting that plastivore enzymes result from exaptation rather than traditional evolutionary processes. This underscores the need for efficient bio-cycling solutions.

### 2.2. Bugging out Plastic: History of Plastic Consumption by Insects

**Figure 2:**
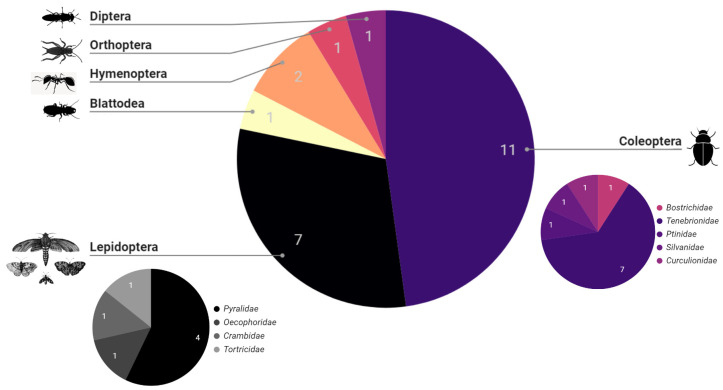
Taxonomic distribution of plastic-consuming insects reported (Table 1). Labels are for the corresponding taxonomic order and sub-labels of the smaller pie charts are for the taxonomic families of Lepidoptera and Coleoptera orders.

Insects first reported as harmful pests causing damage to synthetic polymers in labware, cable insulations, and plastic packaging [16,33], have shown a remarkable ability to consume and break down plastics, with several reported plastic-consuming insects on the rise. Through manual and bibliometric curation, we have identified 23 insect species to be involved in plastic consumption or degradation either through the action of gut microbiota or independently [18,34]. Some of the related species and a reviewed publications summary can be visualized in Table 1. Notably, most of these plastic-degrading insects belong within the orders of Coleoptera (beetles) and Lepidoptera (mainly moths) (Figure 2). Following in predominance are the orders of Hymenoptera, Diptera, Orthoptera, and Blattodea, though unlike Lepidoptera and Coleoptera such orders are not extensively studied for biodegradation (Figure 2 and Supplementary Materials Table S2), [18,35]. This is even though Cleopatra has only 310 publicly available reference genomes on the NCBI database, compared to 1198 Lepidopteran genomes available. The historical milestones in the literature regarding insect-mediated plastic consumption and degradation can be observed through a chronological progression of reports Table 1. Initially, studies focused on documenting instances of plastic consumption by insects. Subsequently, research shifted towards investigating the ability of insects to degrade plastics, indicating their potential role in plastic waste management. Finally, attention turned towards understanding the microbial dependence of insects in plastic degradation and characterizing the enzymes involved in these processes from insects or their symbiont microbes.

There is a need to differentiate between three primary factors pertaining to plastic breakdown facilitated by insects. Firstly, whether breakdown involves true mineralisation or solely physio-chemical fragmentation. In the context of plastic degradation, mineralisation should lead to the incorporation of carbon from the plastic polymer into insect biomass and the evolution of carbon dioxide. However, biotransformation involves the biochemical alteration of pollutants without complete mineralisation. Biotransformation may occur when the plastic molecules are not fully mineralized but rather transformed by redox reactions into other recalcitrant chemicals. Distinguishing between mineralisation and biotransformation is crucial for understanding the extent to which plastic is incorporated into biomass and how various chemical transformations contribute to its degradation in the insect. Accordingly, we highlight the shift in the literature focus for insects as potential solutions to the plastic problems rather than a problem per se in Table 2.

Secondly, it is essential to determine the underlying mechanisms of breakdown, specifically whether it relies on symbiotic microbiota, insect enzymes, or a synergistic interplay of both factors. Insects, particularly those adapting to a diet inclusive of plastic, often harbor symbiotic microbiota within their digestive systems [19,54]. These microorganisms contribute enzymatic capabilities, effectively breaking down the intricate polymer structures of plastics. Concurrently, the insects themselves produce a suite of enzymes, including lipases and esterases, which may play a crucial role in initiating chemical reactions essential for plastic degradation. Deciphering the roles of these components is fundamental in comprehending the intricate dynamics of insect-mediated plastic degradation, offering valuable insights for the development of sustainable strategies to mitigate plastic pollution. Lastly, it is vital to assess the actual extent of degradation, as numerous reports claim plastic degradation but often at negligible levels [55]. The historical development of research involving plastic-eating insects, along with pertinent dates and relevant literature, can be summarized as significant milestones, as depicted in Table 1 and Table 2. Earliest reports of plastic-eating insects emerged in the 1940s but never focused on whether these polymers have been molecularly degraded [16]. 

### 2.3. Exaptation in Plastic Biodegradation: Mechanisms and Polymer Similarities 

#### 2.3.1. Natural and Synthetic Polymers Similarities: Preconditioning Substrates 

The discussion on the similarities between natural and synthetic polymers is not a recent topic. One of the early reports on this subject was published in Nature by Calvert in 1975. The report highlighted the molecular structure similarities between natural and synthetic polymers, using examples such as polyethylene and natural polysaccharide polymers [56]. One of the first attempts at semisynthetic plastic production was cellulose-based collodion in the 1800s [57]. Later, scientists confirmed that collodion removers degrade synthetic plastics [58]. Synthetic plastics and lignin exhibit numerous commonalities in their chemical and physical features, such as non-phenolic aromatic rings, ether linkages, carbon backbone, and hydrophobicity [59]. Due to these shared attributes, lignin-modifying non-specific enzymes such as laccase and manganese peroxidase demonstrated efficacy in degrading synthetic plastics like polyethylene (PE) and polypropylene (PP) [60]. This action has been reported to be an outcome of non-specific radical oxidation [61]. Beeswax is not only one of the natural feeds for the plastic-degrading Greater and Lesser wax moths [43,44] but also a natural alternative to petroleum waxes with prominent levels of chemical and physical similarities [62,63]. Beeswax contains high molecular weight linear hydrocarbons which are structurally similar, albeit with carbon chain lengths that are minor compared to polyethylene [64]. Other natural waxes, originating from plant cuticle and animals for example, do share similar commonalities with synthetic plastics in chemical and physical properties [64].

Chitin stands as the predominant amino polysaccharide polymer found in the natural world, serving as the structural foundation that fortifies the exoskeletons of crustaceans and insects, as well as the cell walls of fungi [65]. Studies have found that chitin not only shares a lot of the chemical and physical similarities with synthetic plastics, but it has also improved oxygen permeability and water vapor properties compared to PE and PS [66]. Keratin is also chemically and physically similar to synthetic polymers and is often used as a bioplastic alternative for the same reason [67]. Moreover, it is also resilient to degradation [68]. 

#### 2.3.2. Wood to Plastic: Termites and Ants’ Appetite for Lignocellulose-like Polymers

Termites are natural wood pests, capable of breaking down usually indigestible natural polymers like cellulose, lignin, and hemicellulose [69]. A commonality between the reported species is being active decomposers or herbivores with a known ability to degrade complex natural polymers. Plant cell walls consist of a defensive network including lignin, cellulose, and hemicellulose. This lignocellulosic biomass comprises 70% polysaccharides and 25% non-polysaccharide polymers [70]. Lignocellulose fibers form physicochemical protective barriers to plants, thus making it the most abundant biopolymer on earth [71]. This depolymerization event facilitates the enzymatic accessibility of complex polysaccharide polymers by hydrolases, thereby enabling subsequent degradation [72]. More studies are crucial for the furthering of potentials, using lignolytic enzymes on synthetic polymers. A variety of termite and ant species have shown the ability to degrade lignocellulose [73]. Termites can digest lignocellulose at ordinary temperatures, unlike herbivorous mammals. Their digestion process is highly effective, with typical bioconversion rates exceeding 95% within a day, facilitated by the breakdown of cellulose (74–99%), hemicellulose (65–87%), and lignin (5–83%) [74]. Transcriptomic analysis reveals that termite host tissues produce a multitude of cellulases, hemicellulases, and lignin-degrading enzymes, thereby aiding in lignocellulose degradation [75]. Table 1 presents various reports documenting the consumption of plastic by termites and ants. The ability of termites to degrade plastic implies that the non-specific enzymatic mechanism employed for breaking down plant-based resilient lignocellulose might be exapted for synthetic polymers [74]. Consequently, herbivorous insect hosts represent a promising source enzyme for depolymerizing synthetic plastics, potentially originating from preconditioning on these natural polymers [76]. 

#### 2.3.3. Plastivorous Moths and Beetles: Beeswax, Chitin, and Keratin 

In 1965, researchers first reported moth larvae damaging polyethylene (PE) and polyvinyl chloride (PVC) [37]. Since then, numerous similar reports have emerged following the findings presented in Table 1. Moths primarily consume natural polymers such as keratin and chitin as a feed source, as evidenced in studies by Shannon et al. (2008) which revealed the presence of keratinolytic bacteria in the midguts of these two moths, aiding in the breakdown of natural polymers such as hair, fur, and decaying matter [77]. The greater wax moth was reported to chew through plastic as early as 1976 [38], preceding recent reports by over four decades [43]. Greater wax moths have shown an unexpected ability to digest synthetic polymers, highlighting their adaptability and potential exaptation of responsible enzymes [43,49]. Termites and ants, like moths, have evolved mechanisms to digest various natural polymers such as keratin, chitin, and lignocellulose and utilize them as carbon sources. This stems from their natural habitat and feeds as decomposers, herbivores, or omnivorous organisms [43,77,78]. Enzyme families expected to be involved in beeswax digestion have also been demonstrated to play a crucial role in PE plastic degradation, namely, lipases 1,3 and carboxylesterases [20]. Chitinase enzymes have been found in many omnivorous and herbivore insects [79] and reported to degrade a variety of plastic polymers [80,81]. Interestingly, keratin also constitutes a major portion of the diet of moths and beetles damaging clothes and textiles [82].

#### 2.3.4. Survival and Developmental Impact of Plastic Consumption on Insects

Studies for insect-mediated plastic biodegradation have been mainly on larvae, as the highest feeding stages of these insects (Table 1), evident by the 7th instar larva of the greater wax moth having the ability to consume a staggering 515.4 ± 22.7 mg of honey wax/day [83]. For comparison, the highest achieved larval weight of *G. mellonella* with a modified diet was less than 400 mg [84]. The physiological impacts on insects consuming plastic are, however, not as agreeable. Studies show conflicting findings depending on the insect species, plastic type, and study duration. When given PVC as their only diet, *T. molitor* larvae had a survival rate of up to 80% in five weeks and as low as 39% in thirty days. When *T. molitor* was fed PVC and wheat bran combined, they achieved development and pupation in 91 days or less, PVC can be broken down by *T. molitor* larvae; however, PVC can only mineralize to a certain extent [55]. Another study spanning 35 days, *Z. atratus* and *T. molitor* were fed PS or PU foam plastic by Wang et al., with bran serving as the control substance. Although, *Z. atratus* had a 100% survival rate, after 20 days of exclusively consuming plastic, there was a decrease in body weight [50]. On the other hand, *T. molitor* survival percentages in the PS and PU groups were 84.67% and 62.67%, respectively, and both groups’ weights rose [50]. Co-feeding impact was also assessed in a study by Lou et al. (2020), showing that PS + beeswax had highest survival (77.3%), over 26% and 228% higher than PS and beeswax alone. PE+beeswax showed similar results, with combined effect much more beneficial to survival than either alone [85]. Survival increased but feed consumption decreased with beeswax or bran added separately to supplement PS/PE. Particularly, beeswax significantly boosted survival of PS-fed (23.6–77.3%) and PE-fed (35.3–66%) larvae [85].

#### 2.3.5. Mechanisms of Insect-Mediated Plastic Degradation

Before consumption of plastics by insects, it is exposed to various abiotic stresses including heat, weathering, and UV radiation which have a significant impact on how easily insects and its gut bacteria break down these polymers [49]. Followed by chewing action, by the insects which in turn increases the surface area available for further enzymatic depolymerization [43]. Plastic fragments’ long chains are then subjected to depolymerization into oligomers because of hydrolysing or oxidative enzymes [18,49]. It is thus, very important to understand the structural differences between different plastic polymers as different bonds require different enzymes. This also explains why some polymers are more resistant to degradation than others, for instance; PE has a very stable linear carbon backbone compared to the ester bonds of PET [51]. This also explains why certain insects have activity towards certain types of polymers (Table 1).

Depolymerization abilities of insects vary between different insects, with some insects depending on gut microbial enzymes for the process, as shown in *Plodia interpunctella*’s dependency on intestinal *Enterobacter asburiae* for PE degradation [50]. While others are independent on gut microbiota in the process as in *Galleria mellonella* for PE degradation [43]. In other cases, it is a mix of dependency and independency as seen in *T. molitor*’s microbial dependence for polypropylene (PP) and PS degradation, but independence for polyethylene (PE) [19]. Plastic-depolymerizing enzymes from insects are yet to be extensively studied. This is evident by the fact that, the only insect-origin enzymes characterized for plastic degradation are four hexamerin proteins reported in the greater wax moth in 2022, named; Demetra, Ceres, Cora and Cibeles [49]. These enzymes are oxidases in nature and have been shown to possess promiscuous array of functions, thus adding more evidence to neofunctionalisation of enzymes [22]. The enzymes responsible for the plastic degradation capabilities of the rest of insects remains to be identified, thus making this a high potential field of research. The last step in the biodegradation is the mineralisation of carbon from the digested oligomers into the biomass and evolution of carbon dioxide. To clearly confirm complete biodegradation of plastic by insects, the incorporation of such carbon in the biomass is vital as demonstrated by Yang et al. (2015) [42].

#### 2.3.6. Factors Affecting Plastic Biodegradation of Plastics

There are multiple factors affecting the biodegradability of a plastics, these factors can be divided into extrinsic and intrinsic factors to the polymer itself. Intrinsic factors such as, crystallinity, molecular structure and weight, additive content, and composition [55,62]. These intrinsic characteristics substantially dictate a plastic’s susceptibility to specific degradation pathways like biodegradation, as they determine features such as enzyme accessibility, moisture absorption and structural stability of the polymer chains. In general, plastics are most conducive to degradation when composed of lower molecular weight, amorphous polymers bearing hydrophilic groups without obstructive chemical substituents or physical architectures [50,55,86].

Generally, polyester based plastics like PET and polybutylene adipate terephthalate (PBAT) have ester functional groups in their structures, that make them more prone to biological attacks as the C-O bonds have less energy and thus less stable than C-C bonds [50,86]. Also, because of the availability of ester polymers in nature as discussed. From natural cutin to beeswax. This also explains, the abundance of discovered microbial PETases in literature (81 hit in the plastic DB database) [87], most of which are cutinase or lipase in nature, which emphasizes on neofunctionalisation. Yet, carbon backbone polymers such as polyolefins (PE, PS and PP) are more resistant to such attacks, due to the strong stability and higher energy of C-C and C-H bonds. Also, scarcity of such bonds in nature rendered organisms less exposed to it and so, there is no natural need for such degradation (2 hits only in plastic DB) [87]. The fact that insects are reported to degrade such recalcitrant polyolefin polymers (PS and PE) independent on microbiota must be fully investigated to detect the intricate factors at play. We view insects as cheap, sustainable, and potentially efficient solution to the plastic problem as it harbors a unique combination of factors at play; physical: chewing, chemical: oxidative and hydrolytic enzymes and biological symbiont bacteria. Moreover, it has been extensively exposed to similar bonds to some plastic types as mentioned in their natural feed [43].

Several key extrinsic environmental conditions can impact the rate of plastic biodegradation. Temperature often correlates positively with degradation potential; however, specialized microbes in certain climates may still efficiently break down polymers at lower temperatures [49,86]. Salinity and pH levels normally found in natural habitats likely exert minor effects. Ultraviolet exposure can aid degradation by oxidizing and fragmenting plastics into smaller pieces more amenable to attack. Overall, degradation proceeds most rapidly under warm, humid, aerobic conditions, allowing optimum activity on hydrated plastic substrates [55,88].

## 3. Bibliometric Materials and Methods

### 3.1. Data Acquisition

The Clarivate™ Web of science (WoS) database was used for the bibliometric analysis data acquisition as it is a highly dependable and inclusive database for this study type and used extensively for a variety of plastic degradation studies [89,90]. Accordingly, we mined the database for articles related to insect-mediated plastic degradation by using a systematically tailored search strategy to eliminate potential false results. The retrieval date and time for the data were 12 February 2024 and 4:12 p.m. (GMT + 8).

### 3.2. Search Strategy

To achieve inclusive yet refined results, we used the following query:

TI = (((“biodegradation” OR “*degradation” OR “ mineralisation” OR “*degrad*” OR “depolymerization” OR “biodeterioration”) AND (“*plastic*” OR “polymer” OR “*polyethylene” OR “polystyrene” OR “polypropylene” OR “polyethylene terephthalate”) AND (“insect” OR “moth” OR “larva*” OR “termite*” OR “ant” OR “beetle*” OR “*worm*” OR “mellonella” OR “molitor” OR “castaneum” OR “interpunctella” OR “grisella” OR “obscurus” OR “cephalonica” OR “confusum” OR “atratus” OR “davidis” OR “dominica” OR “postvittana” OR “nymphaeata” OR “pseudospretella” OR “destructor” OR “brevis”))). The query allows for three mandatory main keywords covering the topic and ensuring refinement based on alternative keywords for the main ones.

### 3.3. Data Analysis

The data mined from the WoS database were used to retrieve a citation report for the results, together with exporting related data such as countries, languages, authors, citations, and source journals. Results were filtered to include only articles, reviews and book chapters avoiding retracted publications and early access papers. Results were reviewed for refinement check without removing any results. VOSviewer software version 1.6.20 was used for the generation and visualization of bibliometric networks. Search results can be found in Tables S3 and S4, Supplementary Materials.

## 4. Bibliometric Results

**Figure 3:**
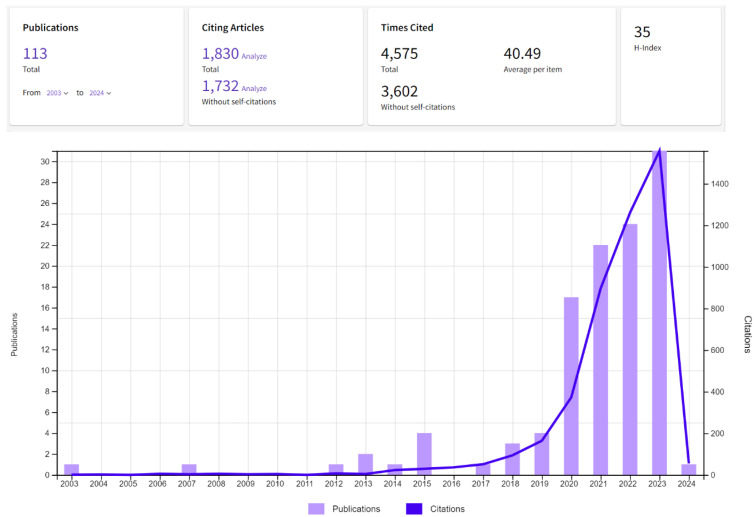
Annual number of publications and the corresponding citations associated with the bibliometric search query for plastic consuming insects as detailed in the methodology section. The *x*-axis represents the year of publication, while the *y*-axis represents the count of both publications and citations.

In total, 113 publications dated between 2003 and 2024 were retrieved from the WoS database using the search strategy in Section 3. Citation details, including publications per year, can be visualized in Figure 3. A total of 12 of 113 publications are labeled as highly cited by WoS scoring a top 1% place among the scholarly discipline of Environment/Ecology, within the field and the year of publication. A significant increase in both citation and publication numbers could be visualized clearly in the last decade Figure 3, with the number of relevant publications jumping from a single publication in 2003, to 31 in 2023. Citations increased from a single citation in 2003, to 1558 in 2023, highlighting the exponential interest in the field and increased scientific effort targeting the plastic waste problem. The top-cited publication presents the work of Yang et al. (2014) titled “Evidence of Polyethylene Biodegradation by Bacterial Strains from the Guts of Plastic-Eating Waxworms” [41]. Considering publications per country, China can be found at the forefront, followed by the USA and South Korea (Figure S1, Supplementary Materials). Note that China being at the forefront aligns with its position as one of the largest plastic producers globally. This likely drives significant research interest in mitigating plastic waste issues domestically through research and other mitigations. In 2016, China imported over half of all plastic scrap shipped internationally. However, this considerable amount sharply decreased by 2018, dropping to under 1% through legislations change [7]. Most studied insect species in China-origin papers is *T. molitor* (43% of China papers), followed by *Z. atratus* (16%). The least studied on the other hand are *A. grisella*, *T. castaneum* and *R. dominica*, each amassing less than 2% of publication occurrences. USA-originating papers showed a similar pattern, with *T. molitor* as the most studied plastivore insect (59% of USA papers), followed by *Z. atratus* (15%) and lastly *G. mellonella* (3%).

**Figure 4:**
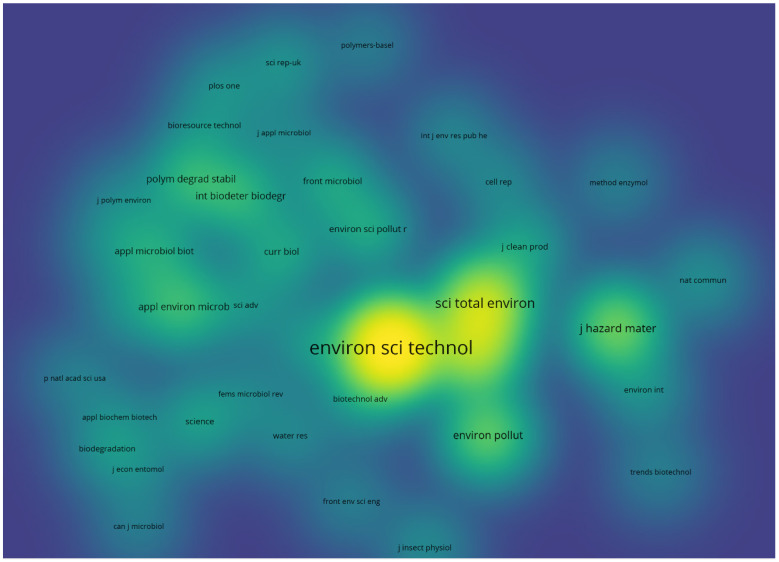
Density map illustrating the distribution of co-cited journals publishing topics related to the query results scope. The association strength normalization method is employed to determine the density. The color of the density map indicates the number of citations, ranging from blue (lowest), green (medium), to yellow (highest). The clustering of journals is based on the link strength of co-cited articles.

**Figure 5:**
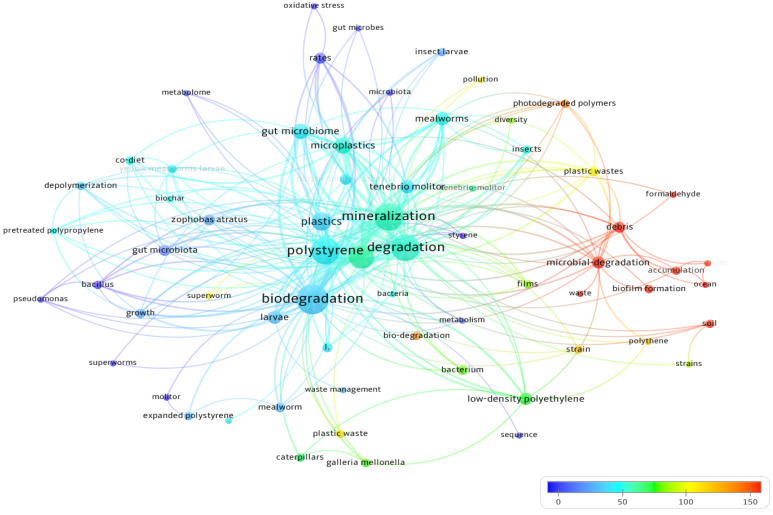
Co-occurrence analysis for all keywords involved in the query results. Color coded based on citation number. Weight is denoted based on the number of occurrences and the linkage of clusters is based on the number of co-occurrences.

Co-citation analysis for all sources (i.e., original publications including cited references) using a cutoff threshold of 10 citations per source, resulted in 40 journals out of 616 total sources, with the most highly cited journal being ACS Environmental Science and Technology, amassing 300 citations. Following closely behind are Elsevier’s Science of the Total Environment with 139 citations and Chemosphere with 116 citations (Figure 4). This analysis could provide relevancy guidance for journal selection. Co-occurrence analysis using keywords occurring three or more times was conducted (Figure 5). Linkages between keywords were set at a threshold of three or higher, with weights based on occurrences.

The top co-occurring keywords were “biodegradation”, followed by “mineralization” and “polystyrene”. Thus, it is crucial to define the extent to which, plastic breakdown is measured. Also, it sheds light on the research favorability of certain polymers. The most common insect keywords were “mealworm” (“*Tenebrio molitor*”) and “*Zophobas atratus*”, followed by “*Galleria mellonella*”. The leading plastic types mentioned were “polystyrene”, then “polyethylene” and “low density polyethylene”, even though polystyrene production is significantly less than the other polymers. This might be attributed to the more recalcitrant nature of polyethylene polymers to degradation. Overall, microbially intermediated degradation appears to be highly cited in these papers, highlighting the importance and the involvement of gut microbiome in these reports and the biodegradation. The highest cited plastivore key word species is “superworm”, followed by “*Galleria mellonella*” and “mealworm” (Figure 5). Clustering of certain insect species keywords with gut microbiota could be clearly visualized, attributing the plastic degradation activity to certain symbiont bacteria as *Zophobas atratus* clusters with superworms (*Z. morio*) and keywords related to bacterial symbionts as “gut microbiota” and “bacillus”. Overall, this analysis provides insight into the research direction focus on certain plastic types, insect species and keywords.

## 5. Discussion and Conclusions

Numerous reports have proposed that the biodegradation of plastics is facilitated by the evolution of enzymes originally intended for breaking down natural polymers like lignin and cellulose [91,92]. However, the presence of plastic-degrading organisms before the widespread production of plastics suggests that exaptation is the more accurate term. This gains further support from studies demonstrating the degradation of newer plastic variants, such as PE and PLA, by different cellulase enzymes [93,94]. Additionally, various lignolytic enzymes have been shown to play a crucial role in plastic degradation [95,96]. This is emphasized by the ability of a wide range of higher species to degrade plastic independent on microbiota [32] making evolution the least accurate term.

The ability of insects to degrade natural polymers like lignin and cellulose, coupled with their reported role as plastic degraders, strongly suggests that this process is a result of exaptation or neofunctionalisation. This is particularly noteworthy considering that many of these insects have long been recognized for their specialized digestive systems capable of breaking down lignin and cellulose [57,97]. Furthermore, the earliest reports of plastivore insects date back to the 1940s [16], predating the widespread commercial production of many plastics. Therefore, exaptation emerges as the most plausible hypothesis for their plastic degradation capabilities. However, it is important to note that the gut microbial communities also play a role in the degradation process, albeit depending on the type of plastic being digested. For instance, *T. molitor* exhibits microbial dependence for polypropylene (PP) and PS degradation, but shows independence for polyethylene (PE), exemplifying the influence of microbial interactions [19].

While the bibliometric analysis retrieved a relatively small number of references (*n* = 113), most publications were published within the last decade, underscoring the recent growth in this research area [89]. A broader search by Akinpelu and Nchu retrieved over twice as many papers (*n* = 290) due to their expanded strategy covering all plastic-degrading organisms rather than solely focusing on insects [89]. This suggests insects have only recently gained attention as plastic degraders compared to microbes, which are extensively reported in dedicated databases like plastic DB (*n* = 753 microbial taxa) [87].

Bibliometric analysis shows the exponential increase in interest for plastic degradation mediated by insects or their gut microbes with most publications originating from China and USA. Also, shedding light on the exponential increase on citations and publications related to the topic. More specific insights are concluded as well, related to the relatedness of specific journals to the topic of insect-mediated plastic degradation. Certain plastic polymers, insects’ species and keywords seem to be more abundant than others in literature. Thus, we aimed to introduce a multitude of historical reports of plastivore insects to enrich the potentiality of studying those as solutions to the plastic problem. We present an extensive dissection of literature reports using manual and bibliometric curation concerning the topic of plastic degradation through insects and consolidate reported species involved in such reports. These plastivore insects provide a potential solution to the plastic waste problem, given their remarkably high rates of plastic consumption [17,18]. While the prevalence of plastic consumption is documented for around 23 insect taxa, further ecological context is needed to establish the full diversity of potential ‘plastivore’ species. Further research is needed to elucidate the enzymatic mechanisms of insect-mediated polymer breakdown. Characterizing enzymatic repertoires across insect taxa may uncover novel catalytic activities and inform efforts to improve substrate diversity and turnover of known plastic-degrading enzymes. Optimization of exogenous factors is also important to balance larval hydrolysis efficiency versus physiological impact. Experiments maximizing nutrition while minimizing toxicity hold promise for advancing plastics bioremediation via insects’ innate biochemical potential. Addressing remaining gaps in knowledge of these biochemical and toxicological determinants can enhance understanding and application. In the pursuit of plastic biodegradation, the main vital factor is efficiency, as many of these organisms/enzymes show either limited degradation rates or incomplete biodegradation. Thus, enzyme engineering might facilitate an accelerated pathway for biodegradation by improving naturally occurring neofunctionalised enzymes.

## Figures and Tables

**Table 1:** History of literature milestones of insect-mediated plastic consumption and degradation. The chronological progression of reports can be observed starting from plastic consumption, then moving on to degradation, and finally investigating microbial dependence and enzyme characterization.

Species	Report	Polymer Type/s	Reference
*Cryptotermes brevis*	Damage	5% Polymethyl methacrylate (PMMA)	[16]
Unspecified (Coptotermes, Heterotermes and Reticulitermes)	Damage	Polyethylene (PE), Polyvinyl chloride (PVC), Neoprene and Rubber	[36]
*Monomorium destructor* & *Camponotus* spp.	Consumption	PE, PVC PE	[33]
*Hofmannophila pseudospretella Stainton &* *Elophila nymphaeata*	Consumption	PE, Polystyrene (PS) and Nylon PE	[37]
*Galleria mellonella and Epiphyas postvittana*	Damage	Unspecified Plastic Containers	[38]
*Rhyzopertha dominica &* *Tribolium castaneum*	Penetration	PE, Polypropylene (PP) and Polyethylene Terephthalate (PET) PE	[39]
*Plodia interpunctella*	Penetration	Unspecified Plastic Packaging	[40]
*Plodia interpunctella*	Breakdown by gut bacteria isolate	PE	[41]
*Tenebrio molitor*	Breakdown (Dependency not assessed)	PS	[42]
*Galleria mellonella*	Breakdown (Dependency not assessed)	PE	[43]
*Achroia grisella*	Breakdown (Dependency not assessed)	High-Density Polyethylene (HDPE)	[44]
*Tenebrio molitor &* *Tenebrio obscurus*	Breakdown (Microbially dependent)	PS	[45]
*Corcyra cephalonica*	Breakdown (Microbially independent)	Low-Density Polyethylene (LDPE)	[46]
*Tribolium castaneum*	Breakdown (Microbially dependent)	PS	[47]
*Tribolium confusum*	Breakdown (Dependency not assessed)	PS, PE and Ethylene-Vinyl Acetate (EVA)	[17]
*Zophobas atratus*	Breakdown (Dependency not assessed)	PS	[48]
*Plesiophthalmus davidis*	Breakdown (Microbially dependent)	PS	[36]
*Tenebrio molitor*	Breakdown (Microbially independent)	LDPE	[19]
*Galleria mellonella*	Breakdown (Microbially independent salivary enzymes characterized)	PE	[49]

**Table 2:** Recent publications suggest a shift in the literature focus to insect-mediated biodegradation as a solution, rather than plastic damage by insects. Relevant key study findings are summarized.

Species	Study Overview	References
Waxworm (*Plodia interpunctella*)	Waxworms depend on gut microbes to break down polyethylene (PE) as their sole carbon source	[50]
Mealworm (*Tenebrio molitor*)	Mealworms, capable of degrading polystyrene (PS) and other fossil-based polymers, convert 47.7% of ingested PS into carbon dioxide, with gut microbes playing a crucial role	[42,51]
Greater Wax Moth (*Galleria mellonella*)	Extensively studied, greater wax moths demonstrate polyethylene (PE) degradation and microbially independent plastic breakdown potential	[20,43]
Rice Moth (*Corcyra cephalonica*)	Rice moths exhibit microbially independent degradation of low-density polyethylene (LDPE), resulting in a weight loss of 21%, indicating their potential role in plastic waste management	[46]
Lesser Wax Moths (*Achroia grisella*)	Investigated for their potential to degrade high-density polyethylene (HDPE), lesser wax moths contribute to understanding plastic degradation mechanisms	[44]
*Tenebrio molitor* and *Tenebrio obscurus*	Both species are involved in microbially mediated polystyrene (PS) degradation, with reported rates of conversion to carbon dioxide, expanding our knowledge of plastic degradation pathways	[46]
*Tribolium castaneum* and *Tribolium confusum*	Investigation reveals insights into plastic degradation pathways, albeit without fully characterizing responsible enzymes	[17,47]
Black Soldier Fly (*Hermetia illucens*)	The black soldier fly contributes to polyethylene (PE) and polystyrene (PS) degradation, emphasizing the role of gut microbes in plastic breakdown processes	[52]
*Cricket species* (*Gryllus bimaculatus*)	Actively participate in plastic waste degradation, with studies highlighting their ability to degrade polyurethane (PU) foam at a rate of 0.28 consumption (mg)/live weight (g) per day	[53]
Beetle Species (*Zophobas atratus* and *Plesiophthalmus davidis*)	Beetle species showed promise in polystyrene (PS) degradation, with microbial involvement highlighted	[48,54]

## Data Availability

All data are contained in this article or the supplementary file.

## Supplementary Materials

The following supporting information can be downloaded at: https://www.mdpi.com/article/10.3390/polym16121671/s1, Table S1: Consolidating the Data for Mismanaged Plastic Waste / Country and the relevant population of the country; Table S2: Displaying the insect species Identified to consume plastic either with or without characterized enzymes or confirmed biodegradation action. Related taxonomic classifications are denoted accordingly to the family and order level; Table S3: Search results of the query targeting the topic of plastic degradation in insects. Relevant publication details are listed accordingly; Table S4: Validation of Bibliometric References; Figure S1: Showcases the top 10 leading countries based on the number of published articles within the current study topic. Number of the published articles per country is denoted accordingly.
